# Dermoid Cysts of the Coccygeal Region

**Published:** 1888-12

**Authors:** E. J. Beall

**Affiliations:** Fort Worth, Texas


### DERMOID CYSTS OF THE COCCYGEAL REGION.

By E. J. BELL, M. D., FORT WORTH, TEXAS.

[Read at Birmingham, Alabama, Meeting of S. S. and G. Association, December 5, 1888, and contributed to Daniel's Texas Medical Journal, by the Author.]

PATHOLOGICAL histology offers up nothing wholly satisfactory in regard to tumors. What is held respecting their etiology is not based upon exact histological knowledge, but is mainly hypothetical.

There are those who consider tumors as tissue-overgrowth; and define their development as due to causes and conditions which odinarily determine hyperplastic growth. In many instances it can be demonstrated that such conclusions are incorrect; for in such, tumor-growth can be shown, by micrescopic evidence, to be of a different nature than the structures from which they spring, or originate. It may be truly said, that a tumor or neoplasm nearly always differs in construction from the tissue out of which it grows.

Another class of etiologists have inferred that tumors are formations resulting trom inflamatory changes. It can be shown, however, that the entire evolution of neoplastic histogenesis is widely at variance with the formative processes that originate in inflammation. We may thus also exclude traumatism from the genesis of tumors. Experience sustains the conclusion; and when it so happens that a tumor appears to develop from traumatized or inflamed tissues, such a result is so rare it may be considered coincidental, and does not prove that such injury or inflammation sufficed to induce the tumor in tissue primarily normal. Other factors are needed before we can claim that the genesis of tumors is due to traumatic or inflammatory causes alone.

Again, it has been suggested to look to the embryo for the factors of tumor production. When we consider that tumors occur at all ages of life—in the new born babe and in the centenarian—and in tissues without the suspicion of abnormality attaching to them, some other explanation will be pertinent before the embryonic theory can take a strong hold upon the profession.

The beautiful hypothesis of Cohnheim bolsters the embryonic theory, and presents the subject for adoption versus Virchow's ideas, to which I have already alluded. He refers the actual evolution of tumors to factors generated during the embryonic period; but considers development after extra-uterine life has occurred, and progressed to any age, due to the continuance of germinal embryonic cells in the otherwise mature organism. The tumor takes its origin from a delayed, lingering cell which escaped utilization in the normal development of tissue and remained unutilized till some external condition supplied the impetus that induced cell multiplication which resulted in tumor production. He thinks the tumor germs consisting of embryonic cells may be small and elude recognition; be quite unrecognizable among the ordinary anatomical elements of the tissues of the body in which the tumor developed. His definition of a tumor then is: "An atypical new-formation starting in a latent embryonic rudiment."

Much that we know of tumors can be harmonized with the Cohnheim theory or hypothesis. Tumors having structures much alike, and the earlier forming stages of certain tissues lend weight to Cohnheim's views. We know that there is a class of tumors which date their origin to the embryonic stage, and such is perhaps the nature of the teratomata or congenital tumors, to which as a special class, the dermoid tumor belongs.

Cohnheim's argument in sustaining his views is partly predicated upon the observations: that many tumors are hereditary; that many are discovered at birth, or develop in early infancy; that a preference is manifested by tumors for sites, when in the earlier developmental stages some complication of structure occurred, such as places where diverse epithelial formations pass into each other (as lips, stomach, cervix uteri, etc.), or for parts where the evolving process is complex (genital apparatus). In fine, that the general atypical nature of tumors is in favor of the probable correctness of his views. Some will concede that these arguments speak strongly for his theory, and will at least admit the probability of its proper adaptability to certain classes of neoplasms, but not applicable to all tumors.

The theory that tumors have an inflammatory, or traumatic origin is held by many, and number among its advocates some of the more advanced etiologists of the present age. We conclude, however, in reviewing these and other hypotheses, that the future will perhaps develop other ideas that shall present such facts, based upon more mature observation, as will harmonize professional thought in regard to the interesting and important subject of tumor genesis.

In a very late issue of the Annals of Surgery, I have read a review of a volume, written by W. Roger Williams, upon the principles of cancer and tumor formation. Without close attention, Williams' theory would be considered a rehash of Cohnheim's; but such is not strictly the case. In the chapter on reproduction, the author states: "I have arrived at the important conclusion, that the processes of repair and reproduction of lost parts, and the various morphological variations, including bud-cancer and tumor formations, are nothing more nor less than abortive attempts of certain cells to reproduce new individuals. Whence it follows, that the laws of reproduction are also the laws of tumor and cancer formation."

There is still another theory: The microbic. I merely make mention of these two last hypotheses of tumor genesis. How they will be received by the profession, is a problem for future solution.

The dermoid cysts, or tumors, which have been claimed as a special class of teratomata, have many peculiarities in common with the normal skin of the body, but occur in positions where skin formations do not normally belong.

The tumors are of interest to the surgeon, as well as to the gynecologist. The latter may encounter them in connection with the the female organs of reproduction. When combined with the ovary, if very small, they may be distinguished by their contents and by the fact that their walls are thicker, whiter and firmer than such envelopes of the Graafian follicles. The larger varieties of these cysts, in this location, have a firm, fibrous envelope; and the microscope will reveal corium and epidermis, hair follicles, sebaceous and sudoriferous glands; now and then adipose tissue, teeth, bone and cartilage are found.

Both young and old are subject to these tumors; more common to the former class. They may develope slowly, or rapidly; may remain dormant indefinitely. I think development is more rapid in the young, as a rule. In five recently observed examples situated at or near the coccygeal region, development began synchronously with the appearance of the beard and pubic hair—all occurred in male subjects. Do not these growths near the mesial line of the body indicate that such formations are derived from the same rudimentary elements as the skin?

Is their evolution dependent upon aberrant germinal cutaneous cells from the epiblast which have wandered to an abnormal site and then at a later period, when activity is given, by natural causes, to skin function or its hair genesis, have begun to develop after their kind? Perhaps more probable and pertinent to the coccygeal dermoid, is the hypothesis: That no epiblastic germinal aberration occurs; but that the epiblastemic cells are turned inwards during fœtal development, and later on, when extra-uterine life has arrived, and certain stages of physical maturation are completed through the law of tissue evolution, these laws spring the activities that develop the tumors, abnormal only because of unnatural position. The same view, I think, will hold in regard to other dermoids than the coccygeal, which may be classed external also, such as those occurring at the extremities of the eye-brows, those intimately connected with bronchial cysts, etc.

The cysts of the coccygeal region vary somewhat histologically, from those encountered in the ovarian region. Such has been my personal experience. Hair, sebaceous matter, epithelium, fibrous and granulomatous tissue have been found; but neither bone nor teeth except in the true teratomata.

The observation of five cases of coccygeal dermoid cysts (so called from location) induce me to present the following inferences:

If a person who is to become the subject of dermoid cyst after passing the age of puberty be examined, there will, in all likelihood (not invariably), be found a depression, or dimple, at or near the sacro-coccygeal region. This dimple, or depression, may be shallow, and the inturning of epiblastic cells so insignificant that at some subsequent period, when life's activities are exagerated by advancing age, only the development of sebaceous glands will occur, and only the result of an aggregation of sebaceous material will be found—no hair, because no hair follicles are present. Such cases present us with an imperfect development of a dermal cyst, and a more perfect formation of the mesial unition that belongs to the embryonic age.

While doing an operation a few days ago, upon an aged subject, for fistula-in-ano, I observed a pit, or depression, as hereinbefore described. This depression was filled with hardened sebaceous material; but the inturning of the skin elements were incomplete, and only the quasi or partial cyst existed.

I have under observation at this time a lad, thirteen years of age, whose mother I attended at his birth, and upon whom I discovered the pimple, or depression I have described. When the age of puberty shall arrive with this boy, and skin function is energized into activity, and follicles and glands shall assert increased function, I confidently expect a greater or lesser development of such tumor as I here present for your consideration. I often think of the now happy boy, ignorant of what is in store for him when he nears manhood ; thinking, perhaps he will go from "pillar to post," from doctor to doctor, as I have seen in such cases ; the one diagnosing an immature abscess ; another caries or necrosis of sacrum, or coccyx. At last an incision reveals the nature of his trouble when he has had the mental and physical torture that attaches to physical infirmity till he reaches the age of twenty-one to twenty-eight years. This is no overdrawn picture. In more than one instance I have heard the story here indicated.

In the cases which came under my observation, when the subjects were twenty-one to twenty-six years old, I saw this pit, or depression ; obliquely away from the pit and mesial line, several times extending down to the gluteal region, I saw the elevated contour of the tumor. Once or twice, extending near the external edge of the sphincter muscle, I saw the same semi-fluctuant elevation that marked the dermoid tumor. These came under notice when the products of the functional activities of the skin had accumulated at the points of abnormal deviation, which began at puberty, perhaps not noticed until several years thereafter. Seemingly an irritation was consequent upon the accumulation. In one or more cases a probe could be carried from the depression above the cyst cavity below. All ran in a downward, or downward and outward direction. When communication with the air had occurred, by examination or by ulcerous process, pus would be added to the retention products.

I pen these lines not on account of the danger to life, (for observation teaches that this does not exist) but to direct attention to a subject little written about; and that I may perhaps save some fellow-practitioner a degree of mortification that comes of mis-diagnosis; for there is a dearth of literature in regard to dermoid tumors of the coccygeal region.

I was invited upon one occasion, by two intelligent gentlemen of the profession, to witness an operation for caries of the sacrum or coccyx. When I saw the case I observed a dimple or depression, at or near the sacro-coccygeal junction. I suggested that the case was not of caries or necrosis, but that a dermoid cyst was to be incised and curretted. A few monents only were needed to verify the correctness of the diagnosis I had volunteered.

The preceding will show that these tumors may be mistaken for caries or necrosis of adjacent bones. They may be mistaken for abscess of the connective tissue of the coccygeal region; may be mistaken for abscesses denominated ischio-rectal; may be mistaken for fistula-in-ano. I think a case of abscess (?), once upon a time, was brought to my friend, Dr. Wyeth, of New York, that proved to be a dermoid cyst of the ano-coccygeal region. Other conditions, benign or malignant, may be thought present when in truth only a dermoid cyst or tumor is to be dealt with.

My experience leads me to the inference that the condition I

imperfectly present to you is of more common occurrence than the writings in text books indicate.

I might write more upon this subject, but enough has been said to put the hearer on his guard when investigating disease or tumors in the region referred to in this paper.

The treatment will suggest itself: Incise the tumor, curette with a Volkman's spoon, and treat with the most approved methods of antiseptic procedure. Nothing more or less is needed. If one wishes, after thorough removal of the deviated growth, the parts may be approximated with reasonable hope of unition, and more rapid growth than by the former method.

With this brief and imperfect review of the theories held to-day respecting tumors, as I have culled them from the memories of reading, and the clinical inferences presented, drawn from my experience with dermoid cysts of the coccygeal region, I end this paper with stating: That these cysts or tumors have been found in various localities other than that to which I have addressed your attention; but you will not find much more than bare mention made of them, and then, seemingly, as among earlier writers, merely such remarks as will gratify a curiosity attaching to such manifestations of abnormally located evidences of deviated development.

